# Local Light‐Controlled Generation of Calcium Carbonate and Barium Carbonate Biomorphs via Photochemical Stimulation

**DOI:** 10.1002/chem.202102321

**Published:** 2021-08-01

**Authors:** Arianna Menichetti, Alexandra Mavridi‐Printezi, Giuseppe Falini, Patricia Besirske, Juan Manuel García‐Ruiz, Helmut Cölfen, Marco Montalti

**Affiliations:** ^1^ Department of Chemistry “Giacomo Ciamician” University of Bologna Via Selmi 2 40126 Bologna Italy; ^2^ Physical Chemistry University of Konstanz Universitätsstraße 10 78457 Konstanz Germany; ^3^ Laboratorio de Estudios Cristalográficos Instituto Andaluz de Ciencias de la Tierra CSIC-Universidad de Granada Av. De las Palmeras 4 18151 Armilla, Granada Spain

**Keywords:** photo-induced crystallization, calcium carbonate, biomorphs, ketoprofen, photochemistry

## Abstract

Photochemical activation is proposed as a general method for controlling the crystallization of sparingly soluble carbonates in space and time. The photogeneration of carbonate in an alkaline environment is achieved upon photo‐decarboxylation of an organic precursor by using a conventional 365 nm UV LED. Local irradiation was conducted focusing the LED light on a 300 μm radius spot on a closed glass crystallization cell. The precursor solution was optimized to avoid the precipitation of the photoreaction organic byproducts and prevent photo‐induced pH changes to achieve the formation of calcium carbonate only in the corresponding irradiated area. The crystallization was monitored in real‐time by time‐lapse imaging. The method is also shown to work in gels. Similarly, it was also shown to photo‐activate locally the formation of barium carbonate biomorphs. In the last case, the morphology of these biomimetic structures was tuned by changing the irradiation intensity.

## Graphical abstract

**Local irradiation** of a liquid precursor leads to the space‐ and time‐controlled photogeneration of calcium carbonate crystals and biomorphs. The method is based on the photodecarboxylation of an organic precursor, and it was optimized to avoid precipitation of organic matter and pH changes. By tuning the light intensity, the morphology of the resulting biomorphs can be controlled.

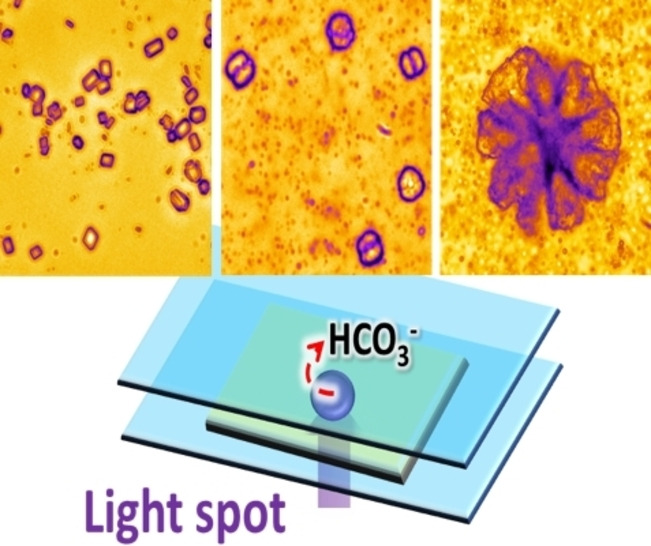

Photo‐activated reactions for the production of solid materials from liquid precursors are at the basis of the most advanced technologies for micro‐ and nano‐fabrication. For photopolymerization, 3D structuring is possible using two‐photon lithography at high speed and with sub‐μm resolution.^[1]^ The main advantage of this approach is that the process can be easily controlled in space and time since the photo‐reaction can be activated only where the light is focused and when the luminous stimulus is switched on.^[2]^ Even though many technologically and scientifically important materials are inorganic, photo‐controlled reactions have been mostly used to produce organic polymeric materials.^[3]^

Focused laser light has been previously used to induce nucleation of supersaturated metastable systems without involving photochemical reactions.^[4]^ In this work, we demonstrate, for the first time, that light‐controlled chemical reactions can be used to locally precipitate calcium carbonate (CaCO_3_), one of the most important inorganic chemical products with many implications in biological, environmental, industrial, and technological processes.^[5]^ Our precipitation technique works well for other alkaline‐earth carbonates and has been successful to promote the synthesis of biomorphs of witherite (BaCO_3_) within local microvolumes of the solution.

Biomorphs are silica‐carbonate curved architectures with hierarchical textures much reminiscent of structural principles found in natural biominerals.^[6]^ They form amazing biomimetic structures easily to identify morphologically, thus offering a dramatic demonstration of the efficiency of our method. Finally, we also demonstrate that our light‐induced precipitation technique also works within agarose gels, allowing the control of the nucleation of metal carbonate crystals in small local volumes where they will grow. This technique opens a gate to the design of three‐dimensional inorganic structures by drawing with light.^[7]^

The chemical basis of the method is simple and clear. A molecular photo generator of CO_2_ is irradiated within an alkaline solution of CaCl_2_. The CO_2_ thus generated converts spontaneously at high pH to bicarbonate and carbonate, thus provoking the precipitation of CaCO_3_ within the area of interest selected by local irradiation with light of a given wavelength. We have chosen the Ketoprofen anion (**K^−^**) as the photoactive molecule in our experiments. This water‐soluble species is known to undergo photo‐decarboxylation, generating in a controlled way bicarbonate that in an alkaline environment is converted into carbonate triggering the precipitation of metal ions as sparingly soluble metal carbonates. The implementation of this idea needs to solve several obstacles that we discuss below.

The light‐activated degradation of **K^−^** anion is schematized in Figure 1. Regarding the mechanism, the absorption of one UV photon leads to the decarboxylation of **K^−^** forming a carbanion which is hence protonated by water. If the photoreaction is performed in a closed system (as the crystallization cell shown in Figure 1), the net process will form a water‐insoluble photoproduct **PP** and the bicarbonate anion. Indeed, the local photo‐precipitation of **PP** was demonstrated using an inverted microscope for focusing UV light, as shown in Figure 1. As discussed in detail in the Supporting Information the formation of HCO_3_^−^ was demonstrated by measuring pH decrease of alkaline **K^−^** solutions during irradiation. We would like to underline that local PP photo‐precipitation and pH drop represent severe drawbacks for carbonate photo‐precipitation since: i) the PP precipitate contaminates the photo‐produced carbonate and ii) pH drop leads to carbonate dissolution.^[8]^ These obstacles were overcome by adding to the solution a Triton X that incorporated and solubilized the PP forming micelles and by using a NH_4_OH/NH_3_ buffer, respectively.

**Figure 1:**
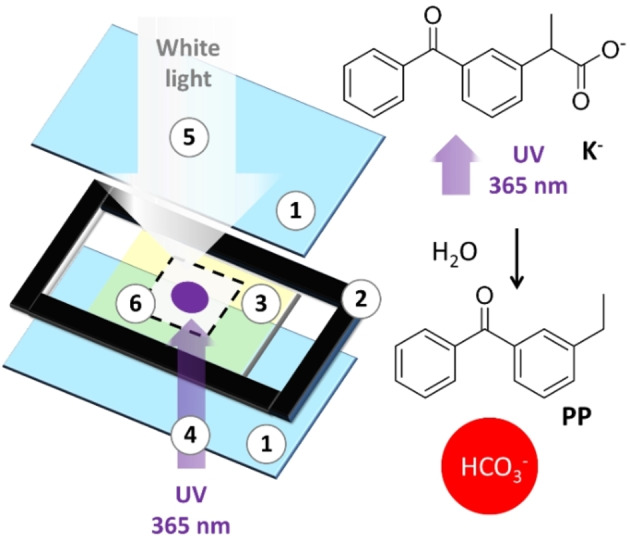
Left: Schematic illustration of the irradiation cell, which is made by two slides of glass (1) separated by a rubber spacer (2) 0.1 mm thick. The cell was filled with the crystallization solution containing **K^−^** (3) and then sealed. UV light of a LED at 365 nm was focused on a 300 μm radius spot (4) in the solution with a microscope objective. A white light illuminator (5) was used to detect the time‐lapsed images of a large area of solution (6) during the UV local illumination. Right: the reaction of photodecarboxylation of **K^−^** which leads to the formation of the photoproduct, **PP**, and of HCO_3_^−^.

Next, the local calcium carbonate photo‐precipitation was investigated with an inverted optical microscope using the optimized solution containing **K^−^** (20 mM), NH_4_OH/NH_3_ buffer (40 mM), and Triton X‐100 (0.3 mM) after addition of CaCl_2_ (20 mM). The experiment was performed upon spot irradiation with a 365 nm diode in the crystallization cell as schematized in Figure 1.

Time‐lapsed acquisition (0.1 frame/s) for 1200 seconds in transmission mode with a 20 MPX CMOS camera allowed the formation of CaCO_3_ to be followed during local (300 μm radius) irradiation using a 10X magnification objective.

As shown in Figure 2a, no crystals could be observed in the detected area before irradiation. Figure 3b–c shows the formation of crystals in the proximity of the irradiation spot. Tiny crystals formation could be detected after 400 s irradiation (Figure 2b). These crystals were observed to grow in time after 800 s (Figure 2c) and after 1200 s (Figure 2d). Note that crystallization occurs mainly in the corresponding irradiated area and that the crystals cover about 5 % of the total surface.

**Figure 2:**
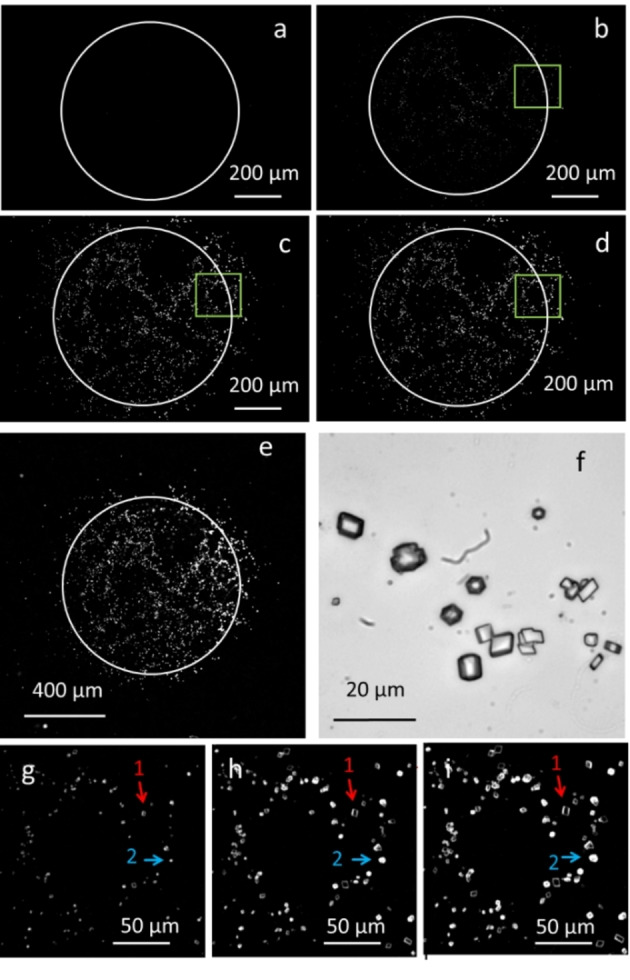
a–d) Transmission images of a solution containing **K^−^** (20 mM), NH_4_OH/NH_3_ buffer (40 mM, pH 8.9), Triton X‐100 (0.3 mM) and CaCl_2_ (20 mM) in a 0.1 mm thick glass cell irradiated with a circular spot of UV light (365 nm, 1.3 mW/mm^2^, shown as a white circle) for 0 s (a), 400 s (b), 800 s (c) and 1200 s (d). Transmittance image after 1200 s irradiation at lower and higher magnification are shown in e) and f), respectively. The green square area shown in b, c, and d was digitally enlarged g, h and i. In b‐d, the simultaneous growth of rhombohedral crystals (as the one labeled 1) and of more spherical crystals (as the one labeled 2) can be followed.

**Figure 3:**
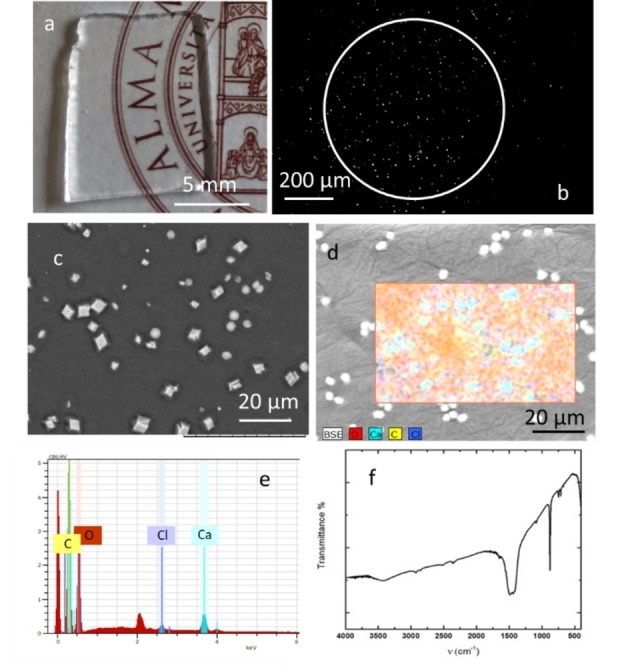
a) Photograph showing the transparency of the agarose gel used for the photo‐crystallization. b) Transmission image of the gel loaded with CaCl_2_ 20 mM, covered with a 20 mM solution of **K^−^** and irradiated with a UV light spot (white circle) for 30 minutes. c) and d) are the SEM image and the EDX image of the crystals obtained after irradiation of the gel after drying. e) EDX spectrum of the area shown in d). f) IR spectrum of the crystals obtained by photo‐precipitation after washing with EtOH and drying.

However, some crystals were formed slightly outside this area, in a band about 100 μm thick, because of the diffusion of the photoproduced HCO_3_^−^ out of the light spot. This local precipitation confined close to the illumination spot was confirmed by observing the exposed areas with a lower magnification objective of 4X to expand the field of view. As shown in Figure 2e, no crystals are observed at a distance farther than 100 μm from the illumination spot. To identify the polymorphs of calcium carbonate by the morphology of the crystals, the green square area of Figure 2b–d was digitally enlarged (Figure 2g–i). In the enlarged Figures 2g, 2 h, and 2i (corresponding to 400 s, 800 s, and 1200 s irradiation, respectively), it is possible to follow the simultaneous growth of rhombohedral crystals (morphology typical of calcite), like the one labeled as 1. In addition, some spherical crystals labeled as 2 are also observed, which is characteristic of vaterite, the hexagonal polymorph of calcium carbonate.

These results demonstrated that the photoproduction of CaCO_3_ can be triggered within a few hundred micrometer region of a water solution in a controlled way. Nevertheless, for the actual application of this approach, the immobilization of the photogenerated crystals in a matrix is undoubtedly advantageous, as it will avoid sedimentation of the formed crystals and decrease diffusion of the formed bicarbonate, thus increasing the resolution of the technique. Therefore, we have performed similar experiments within an agarose hydrogel (modified with sucrose for refractive index matching to increase the transparency) that works as a crystallization substrate^[5c]^. The hydrogel is shown in Figure 3a. It was prepared as described in the Supporting Information, having a final thickness of 1 mm, loaded with calcium ions, covered with a solution of **K^−^** (20 mM), and irradiated with the focused UV light spot. After 30 minutes of irradiation, a spot of calcium carbonate crystals could be detected on the transmission image (Figure 3b). To characterize the grown crystals, the gel was dried in an argon atmosphere and analyzed by SEM (Figure 3c) and EDX (Figure 3d). As in solution experiments, calcium carbonate crystallizes mainly as calcite, with fewer vaterite crystals also forming. The concomitant precipitation of calcite and vaterite was confirmed by the IR spectra shown in Figure 3f. The decreased diffusion in the gel can be deduced from Figure 3b. Crystals are found in an area of only 70 μm around the irradiation spot, although the irradiation time of 30 min was 1.5 times longer than the one in Figure 2e.

To demonstrate the general application of our method for space and time‐controlled photo‐stimulated production of carbonate, we investigated its applicability to the photogeneration of biomorphs. Silica/carbonate biomorphs are fascinating inorganic‐inorganic composite nanocrystalline structures that emulate the textures and shape of living organisms.^[8]^ This property facilitates their identification, and therefore they are perfect markers of carbonate precipitation. Silica biomorphs have been reported to form in silicate‐rich alkaline solutions containing alkaline‐earth metals, like Ba^2+^ or Sr^2+^, upon exposure to atmospheric CO_2_.^[9]^ Therefore, in this case, **K^−^** (5 mM) was added to a solution containing sodium silicate (30 mM), BaCl_2_ (5 mM), and NaOH for final pH=11.7. This solution, kept under nitrogen flow, was used as a precursor for the photogeneration of the biomorphs. For the local photogeneration of the biomorphs, we used the closed glass cell, shown in Figure 1 and described above.

After filling the cell with the biomorph precursor solution, time‐lapsed transmission images were acquired at the inverted microscope under local illumination. In the first experiment, the UV light irradiance was the same as the one previously used for the calcium carbonate photogeneration (1.3 mW/mm^2^). The time‐lapsed image sequence showed the growth of the biomorphs after 9000 s only in the UV illuminated spot. The final structure of the biomorphs shown in Figure 4a (magnified with a 60X objective in the inset of Figure 4a) shows the typical dual lobe structure of barium carbonate biomorphs, a fake mitosis‐like morphology, which is the end‐term of the fractal formation route. Indeed, the biomorphs obtained under these irradiation conditions are numerous (per surface area) and quite monodisperse in size. However, they are relatively small. This is consistent with fast nucleation induced by high supersaturation, which is produced by the rapid generation of carbonate from **K^−^** photodegradation under intense illumination. Hence, we investigated the effect of a weaker irradiation intensity by repeating the experiment at an irradiance 0.1 mW/mm^2^. As expected, the sequence of time‐lapse images of this experiment at a slower rate of photogeneration of carbonate revealed that the nucleation density of biomorphs (number per surface unit) is smaller. Consequently, the biomorphs reached a considerably larger size at the end of the irradiation. As shown at the inset of Figure 4b (upon magnification at 60X), these biomorphs also showed a different, more complex morphology when compared to the ones obtained at higher irradiance. By expanding the image sequence acquired at 10X magnification, it was also possible to follow, in real‐time, the growth of an individual biomorph during the irradiation: the different steps of the development are shown in Figures 4c1–c8.

**Figure 4:**
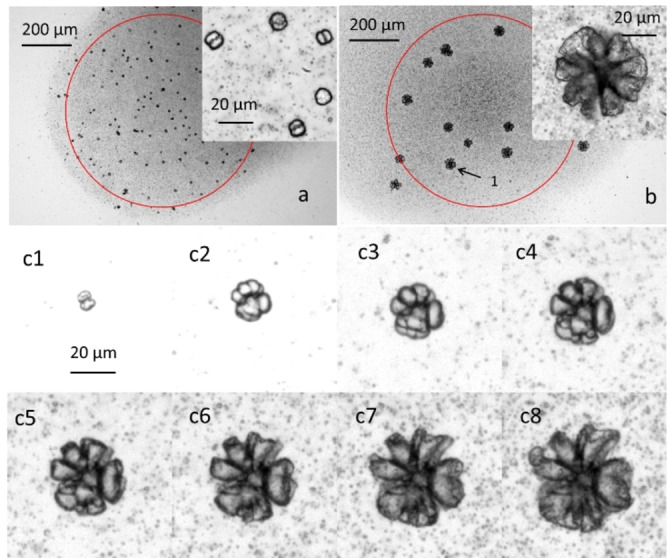
a) Transmission image of BaCO_3_ biomorphs obtained after 900 s irradiation (UV spot 365 nm, 1.3 mW/mm^2^ is shown in red). Inset shows the morphology of the biomorphs. b) Transmission image of biomorphs obtained after 150 minutes irradiation (UV spot 365 nm, 0.1 mW/mm^2^ is shown in red). Inset shows the morphology of the biomorphs. c1–c8) Transmission images of the biomorph labeled as 1 in b after 1000 s, 2000 s, 3000 s, 4000 s, 5000 s, 6000 s, 7000 s, 8000 s, and 9000 s, respectively.

In conclusion, we have demonstrated that the photo‐induced decarboxylation of a molecule like **K^−^** can be exploited to produce HCO_3_^−^ locally and hence, in an alkaline environment CO_3_^2−^. The local production of carbonate induces crystallization of carbonate‐based structures like calcite or biomorphs. Additionally, as shown in Figure 4a and b, in the case of biomorphs, by tuning the irradiation intensity, it is also possible to control, for the first time with a photochemical method, the morphology of the resulting structures. This will allow for the writing of different biomorph structures in the same experiment by varying the irradiation time at different irradiation spots. Also, variation of the irradiation time in subsequent growth cycles might produce carbonate/silica structures with several coexisting textures as was revealed for temperature variations^[10]^ but now with control of the biomorph nucleation site.

Although substantial localization of the crystallization was demonstrated, diffusion of the photoproduced species out of the irradiation spot partially limited the space resolution. However, we would like to stress that the study presented here was aimed to demonstrate the feasibility and the versatility of a new photochemical strategy for photo‐induced crystallization that we believe can be easily extended from carbonate to a wide variety of inorganic materials.

Hence, our approach has significant potential for future applications, and the resolution of the written structures can be enhanced using an appropriate UV laser with corresponding focusing optics rather than the here applied LED. This would enable 2D photolithographic crystallization with precise writing of crystallographic structures just limited by the size of the laser spot and the size of the growing crystal if illumination times can be optimized to be short enough that bicarbonate diffusion is not relevant. For our example in Figure 2, reduction of the illumination time from 1200 s to 1 s by a sufficiently high light intensity would reduce diffusion by a factor of 35 since diffusion scales with the square root of time. Photo‐induced patterned crystallization with a μm sized focused laser beam could, for example, improve the resolution of the recently introduced “Ion Exchange Lithography”,^[11]^ which relies on PbCO_3_ transformed to perovskites with ion exchange reactions by inks, which would not need to be printed anymore with the associated limitations.

Finally, our approach is in principle transferable to other photo‐labile molecules or pH‐dependent reactions and even the application of several photolabile molecules with different chromophores at the same time opening a whole range of photo‐induced (nano)crystals like semiconductors or metals for 2D or even 3D writing with light towards miniaturized electronics or other applications.

## Conflict of interest

The authors declare no conflict of interest.

## Supporting information

As a service to our authors and readers, this journal provides supporting information supplied by the authors. Such materials are peer reviewed and may be re‐organized for online delivery, but are not copy‐edited or typeset. Technical support issues arising from supporting information (other than missing files) should be addressed to the authors.

Supporting Information

Supporting Information

Supporting Information

Supporting Information

Supporting Information
