# Supplementary material for: Local Light‐Controlled Generation of Calcium Carbonate and Barium Carbonate Biomorphs via Photochemical Stimulation
Source: Chemistry. 2021 Aug 1;27(49):12521–5. doi: 10.1002/chem.202102321 (PMC8456953; doi:10.1002/chem.202102321)
Supplement: Supplementary file 1 — Supporting Information [file CHEM-27-12521-s003.pdf]

Chemistry–A European Journal

Supporting Information

Local Light-Controlled Generation of Calcium Carbonate and Barium Carbonate Biomorphs via Photochemical Stimulation

Arianna Menichetti, Alexandra Mavridi-Printezi, Giuseppe Falini, Patricia Besirske, Juan Manuel García-Ruiz,* Helmut Cölfen,* and Marco Montalti*

1 Materials and Methods

1.1 Materials

All reagents, solvents, and chemicals were purchased from Sigma-Aldrich and used without modification unless otherwise stated. In particular, Ketoprofen (101833584), Triton X-100 (x-100), Sodium Silicate (338443), Agarose (1001281256), Sucrose (1076870250) were used.

1.2 UV-Vis Spectroscopy

The experiments were carried out in air-equilibrated solutions at 25 °C. UV–Vis absorption spectra were recorded with a Perkin-Elmer Lambda 650 spectrophotometer using quartz cells with a path length of 1.0 cm.

1.3 Mass Spectrometry

Electronic impact mass spectrometry (GC-MS). GC Agilent Technologies 6850 interfaced with mass spectrometer single quadrupole MSD 5975.

1.4 pH measurements

pH measurements were performed with the use of a pH-meter (Jenway 4330 - Alessandrini).

1.5 Dynamic Light Scattering (DLS)

DLS measurements were performed with Zetasizer Nano ZS Malvern Panalytical using PMMA semi-micro cuvettes (BRAND).

1.6 Microscopy

Microscopy was performed using an Olympus IX 71 inverted microscope equipped with a 365 nm LED for irradiation and a Basler acA5472-17uc camera with a Sony IMX183 CMOS sensor. The sensor size is 13.1 mm x 8.8 mm, while the pixel size is 2.4 μm x 2.4 μm. The UV LED was positioned in the backport of the microscope, and light was collected by a 10X objective after being filtered

(band-pass filter Chroma 350/50x) and 90° reflected by a dichroic mirror (Chroma T400lp). The size of the UV irradiation spot was about 300 μm radius, and it was centered at the centre of the objective. Irradiance in the spot (at 3.9 V and 0.9 A) was 1.3 mW/mm^2. The irradiation was performed with a 10x magnification objective (Olympus UPLFLN 10X), The current intensity was managed by a RS PRO IPS 303DD laboratory DC power supply. For the preparation of the microscopy samples, Aptaca 26x76 microscope slides were used with thickness 1.00/1.20 mm.

1.7 SEM

SEM measurement was performed using a Hitachi TM3000.

1.8 Irradiation Source

For the performance of the experiments, we used:

- LED (3.2 mm ø) purchased from Mouser Electronics (Led Engin LZ1-10UV0R) The irradiation wavelength is 365 nm with a radiant flux of 1320 mW (Forward current 700 mA-1A) at 25°C. The LED has a viewing angle (the off-axis angle from the centreline where the radiometric power is the 50% of the peak value) of 70°.
-

2 Photophysical and photochemical characterization of Ketoprofen and organic by-products

Photo-reactivity of Ketoprofen is well documented [1] [2] ; nevertheless, photoproduction of CO_2 from Ketoprofen (or other organic precursors) has been rarely exploited for the photo-precipitation of Calcium Carbonate or photogeneration of biomorph and never before in a localized way. [3] [4] [5] [6] Since Ketoprofen is not soluble as a carboxylic acid in water solution, we used the deprotonated form **K**$^-$, which is formed at pH>7 (pk$_a$=5.94).

Although ketoprofen and ketoprofen anion show a main absorption band at 270 nm, [7] we preferred to use a 365 nm LED for irradiating since: i) conventional microscopy optics are transparent to this wavelength and conventional microscopy objectives can be used to focus the light in a small spot (300 μm radius with 10X); ii) 365 nm LED's are cheap, widely available high-power light sources,

and they are less dangerous than UVC light sources. iii) **K⁻** can still be excited with 365 nm light as shown in the UV–Vis absorption spectrum of Ketoprofen (20 mM) in water at pH 8.9 of Figure S1.

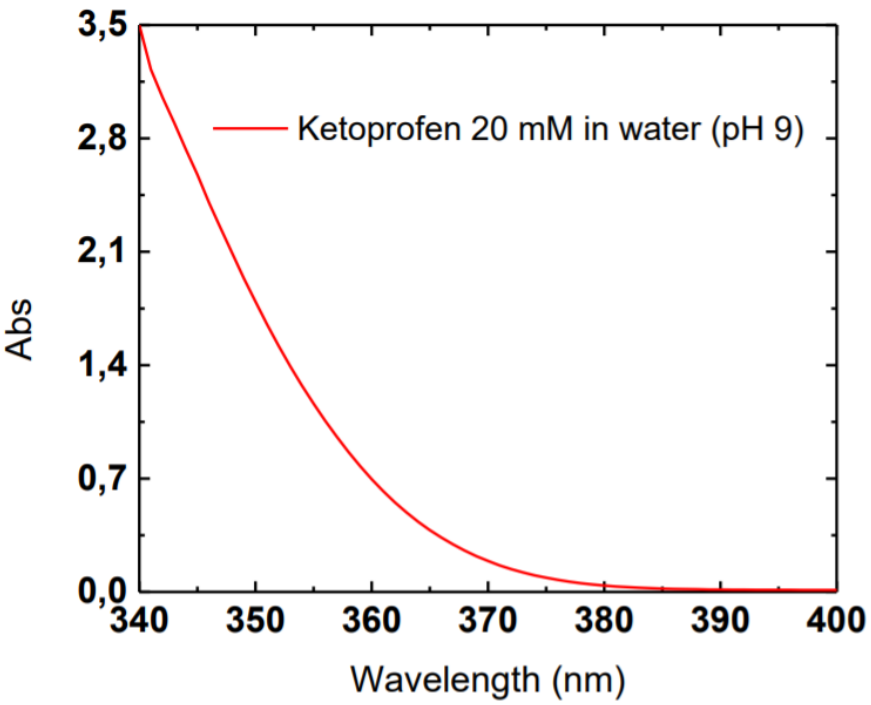

Figure S1. UV-Vis absorbance spectrum of Ketoprofen 20 mM in water at pH 8.9

2.1 Optimization of the photoreactive solution for the photo-precipitation of calcium carbonate

For local irradiation, we used the light from a 365 nm UV LED in a spot with a 300 μm radius (irradiance 1.3 mW/mm^2) in a closed cell with 0.1 mm thickness and 15x25 mm^2 size, as schematized in the main text, figure 1. The cell was filled with a **K⁻** solution of 20 mM at pH = 8.9. and was irradiated for 120 s; during irradiation, the transmission images were acquired with a 20 MPX color CMOS camera by trans-illuminating a large area (about 4x4 mm^2) of the cell, centered on the UV spot, with white light (not absorbed by **K⁻**).

2.1.1 Demonstrating local photo-precipitation of PP

The local irradiation of the sample mentioned above led to the formation of oily droplets of the **PP**. The chemical nature of the **PP** was investigated by gas chromatography-mass spectrometry

(see below), in which a single chromatographic peak characteristic of 3-ethylbenzophenone was observed. The precipitation of the **PP** creates a double problem. First, because of their size, these droplets diffused very slowly outside the irradiation area, remaining in the illumination spot even 30 min of illumination (see Figure S2 a), filtering the UV light, limiting the photoreaction rate, and reducing the generation of bicarbonate.

2.1.2 Demonstrating photo-generation of HCO_3^-

To demonstrate the photogeneration of HCO_3^-, we irradiated with the 365 nm LED a 20 mM solution of **K**$^-$ at pH = 8.9 (obtained by the addition of NaOH) in a closed vial measuring in the meantime the pH with a glass electrode. As shown in figure S2 (black dots), a decrease in the pH was observed during irradiation. Nevertheless, this change was relatively small because of the screening effect mentioned above.

2.1.3 Preventing local photo-precipitation of PP

To avoid the **PP** precipitation, the experiments were repeated in the presence of Triton X-100 (0.3 mM), a surfactant known to form tiny micelles able to solubilize organic molecules in water (the formation of small transparent droplet was demonstrated by dynamic light scattering and UV-Vis spectroscopy as shown below).

As shown in figure S2 c, in the presence of Triton-x 100 (0.3 mM), the local irradiation of the 20 mM **K**$^-$ solution at pH 8.9 at the inverted microscope did not reveal the formation of any organic precipitate, demonstrating the ability of the surfactant to solubilize the **PP**. As far as the pH change during irradiation is concerned, figure S2 b (red triangles) clearly shows a considerable decrease in pH with time, demonstrating the photogeneration of HCO_3^-.

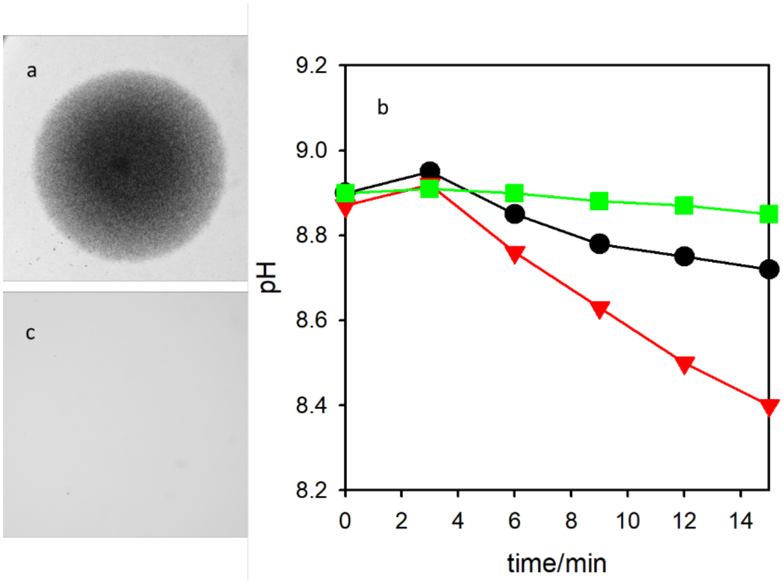

Figure S2. a) Transmission light microscopy image showing droplets of **PP** formed upon local irradiation of a $\mathbf{K}^{-}$ solution 20 mM at initial pH 8.9 (adjusted with NaOH) for 2 minutes. The irradiation spot was 300 μm radius. b) Evolution of pH values during the irradiation with a 365 nm LED of: (black dots) a solution containing $\mathbf{K}^{-}$ at initial pH 8.9 (adjusted with NaOH); (red triangles) a solution containing $\mathbf{K}^{-}$ at initial pH 8.9 (adjusted with NaOH) and Triton X-100 0.3 mM; (green squares) a solution containing $\mathbf{K}^{-}$ (NH_4OH/NH_3 buffer, pH=8.9) and Triton X-100 0.3 mM c) Transmission image obtained in the same conditions as for b) in the presence of Triton X-100 0.3 mM.

2.1.4 Preventing pH drop during the photoreaction

While the solution acidification is a clear indicator of the formation of HCO_3^-, it does not favor the aim of using this photoreaction to precipitate calcium carbonate. The solubility of calcium carbonate is strongly pH-dependent, and it increases at lower pH values. Therefore, an alkaline pH must be secured to trigger the precipitation. Hence, an NH_4OH/NH_3 buffer (40 mM, pH 8.9) was added to prepare a solution containing $\mathbf{K}^{-}$ 20 mM and Triton X-100 (0.3 mM). In this latter solution, as shown in figure S2 b (green squares), a minor pH change was observed during the irradiation.

2.1.5 Mass Spectrum of PP

Electronic impact mass spectrometry (GC-MS) was performed on Ketoprofen 6 mM solution in water/ethanol 1:1 mixture after 1 hour of LED irradiation (Figure S3). The LED was placed horizontally, at 2 cm from the vial.

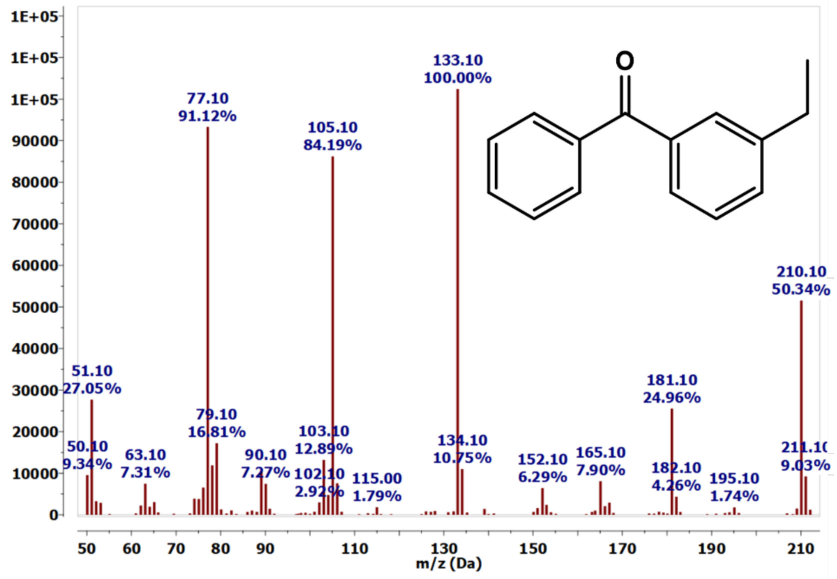

Figure S3. GC-MS spectrometry of Ketoprofen after 1 hour of irradiation at 365 nm. Ketoprofen by-product generated by the decarboxylation process is observed.

2.1.6 Details for pH measurements

The pH change of Ketoprofen (20 mM) solution in water and water with Triton X-100 0.3 mM was monitored during LED irradiation. The LED was placed horizontally, at 2 cm from the vial.

To estimate CO_2 production effect on the overall pH, monitoring was performed irradiating Ketoprofen (20 Mm) solution at pH 9 in two cases: in the presence of NaOH (in water and in water and Triton X-100 0.3 mM) and in the presence of NH_3 (in water and Triton X-100). The change in pH was monitored every 3 minutes for 15 minutes.

2.1.7 Dynamic Light Scattering (DLS) of Triton X-100 and Ketoprofen solution

The size of the nanoparticles formed in the presence of the surfactant, Triton X-100, was investigated via DLS. In particular, a solution of 20 mM Ketoprofen and 0.3mM Triton X-100 in water at pH=9 was

prepared, and the analysis was performed in two different time intervals using PMMA semi-micro cuvettes. The DLS was received after irradiating the solution for 15 (Figure S5b) and 25 minutes with the 365nm LED. The results indicated the presence of nanoparticles with a size around 100nm and narrow size distribution (Figure S5a).

a.

Sample	Z-Average	PDI
K+TX irradiated for 15 minutes (pH9)	103 nm	0,097
K+TX irradiated for 25 minutes (pH9)	110 nm	0,091

b.

			Size (d.nm):	% Intensity:	St Dev (d.nm):
Z-Average (d.nm):	103,0	Peak 1:	115,3	100,0	40,05
PdI:	0,097	Peak 2:	0,000	0,0	0,000
Intercept:	0,943	Peak 3:	0,000	0,0	0,000
Result quality :	Good				

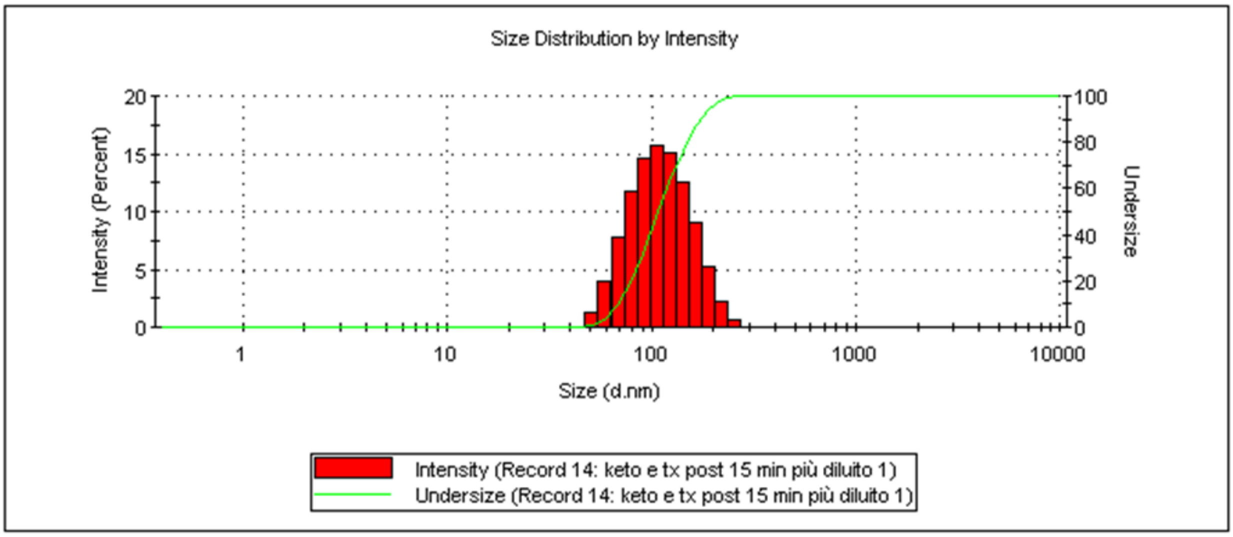

Figure S4. a) Z-Average and PDI (polydispersity index) results of (20 mM) Ketoprofen and (0.3mM) Triton X-100 solution in water at pH=9 after 15 and 25 minutes of irradiation with a 365nm LED received by DLS b) DLS analysis of (20 mM) Ketoprofen and (0.3mM) Triton X-100 solution in water at pH=9 after 15 minutes of irradiation

2.1.8 SEM of $CaCO_3$ crystals formed in solution

Ketoprofen 10 mM and CaCl2 10 mM solution was irradiated by a laser at 355 nm for a few minutes and all over the solution to obtain as much precipitate as possible. Then, the precipitate was collected

and dried. SEM measurements were performed with a Hitachi TM3000 and are shown in Figure S5. In Figure S5, it can be noted that a lot of Calcite crystals are deposited on the organic matrix, which probably corresponds to the water-insoluble Ketoprofen by-product formed after irradiation.

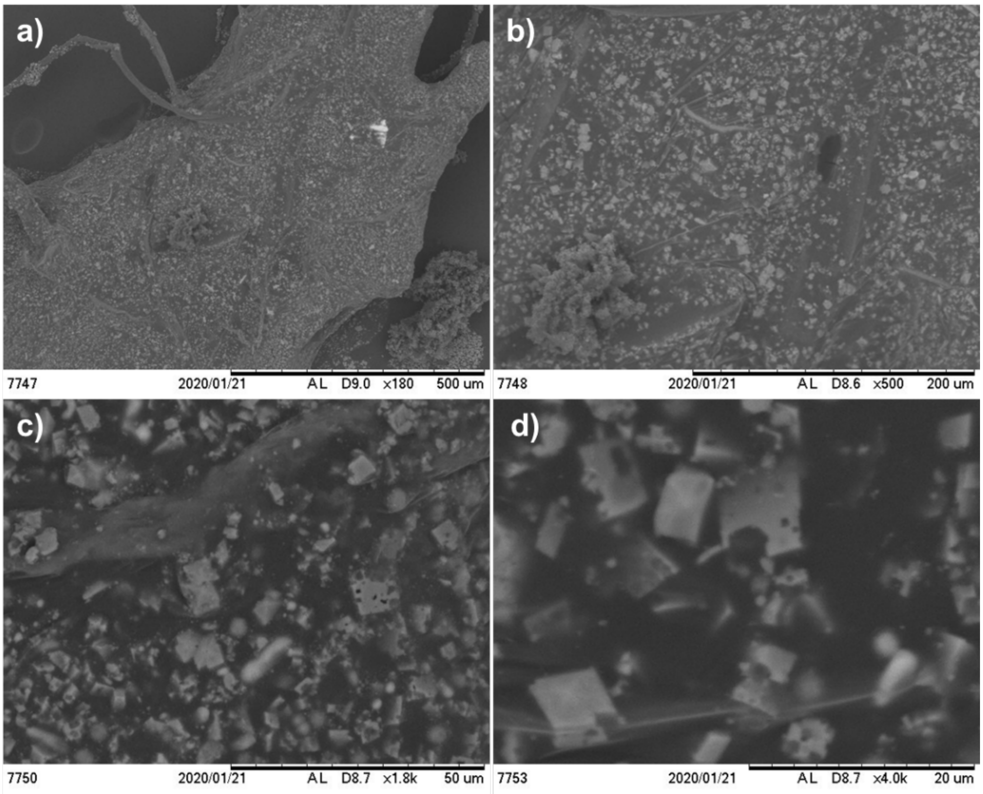

Figure S5. SEM images of $CaCO_3$ deposited on organic matrix after laser irradiation at 355 nm

2.2 Photoreaction quantum yields

Quantum yields (Figure S5) were calculated by irradiation of Ketoprofen 20 mM in water, without and with $CaCl_2$ 20 mM.[8] Irradiation was performed in a fluorimeter (Fluoromax) at 365 nm, with a slit of 20 nm. Almost 3 mL of solution were irradiated under stirring; every 10 minutes, the solution was centrifuged (5 min, 8000 rpm) and filtered twice with a 0.45 µm PVDF filter. Then the absorption spectrum was measured before continuing with the irradiation.

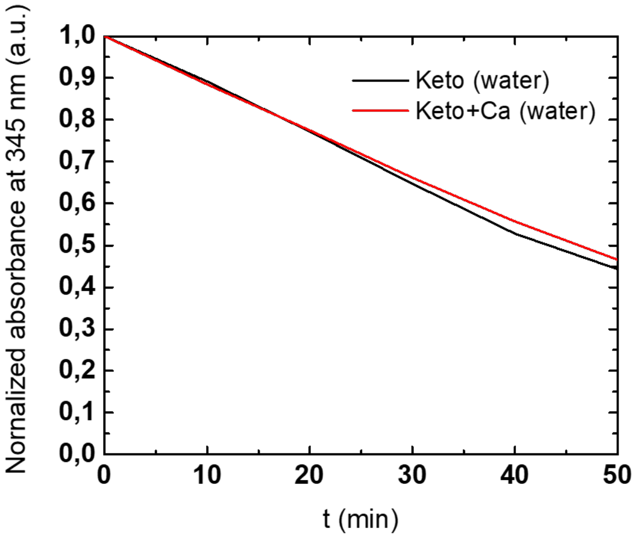

$$\Phi \sim ([R_0] - [R_t]) \cdot \frac{V \cdot N_A}{q_{p,in}} \cdot \frac{1}{(f_{as})_m} = 1{,}5\ \%$$

$[R_0]$= Ketoprofen concentration at t= 0 min
$[R_t]$= Ketoprofen concentration at t = 50 min
N_A= Avogadro number $6.02 \cdot 10^{23}$
V= 0.003L
$q_{p,in} = 0.95 \cdot 10^{18}$, calculated by Potassium Ferrioxalate actinometer

$$(f_{as})_m = \frac{\left(1 - 10^{-A_{0 min}}\right) - \left(1 - 10^{-A_{50\ min}}\right)}{2}$$

$A_{0\ min}$: Absorbance of Ketoprofen at 345 nm at the beginning of the irradiation (0 minutes)
$A_{50\ min}$: Absorbance of Ketoprofen at 345 nm at the end of the irradiation (50 minutes)

Figure S5: Normalized absorbance decrease (left) of Ketoprofen at 345 nm, without (black) and with (red) Calcium Chloride 20 mM. Quantum Yield calculation (right).

The photoreaction quantum yield was calculated based on the Ketoprofen absorbance decrease at 345 nm. This value, which is in the tail of the absorption band of Ketoprofen, whose maximum is at 270 nm, [7] was chosen because the high Ketoprofen concentration did not allow to monitor its absorbance variation at the maximum of the peak. As Figure S5 shows, the quantum yield does not depend on the presence of Calcium Chloride and is 1,5%.

3 Gel preparation

Agarose (20 mg/mL) is added to a sucrose 60% w/v aqueous solution, in presence of $CaCl_2$ 20 mM. The system is heated at 85°C to dissolve agarose. The hot solution is left to cool down between two glass slides to obtain a very smooth and uniform gel with a thickness of 1 mm.

3.1 Photo-precipitation of Calcium carbonate embedded in a gel

A piece of the gel prepared with $CaCl_2$ 20 mM (almost 1cmx1cm) was placed in a disposable well (0.57 cm^2) and covered by 350 µL of Ketoprofen 20 mM solution at pH 9.6. The irradiation is performed using a LED at 365 nm focused by the fluorescence microscope, utilizing a 4x objective**.**

Then, the solution is removed, and the gel is washed with MilliQ water, which is then removed. The formation of crystals in the gel is then observed by the camera, with the 4x objective.

4 Silica–Carbonate Biomorphs

Initially, a solution of NaOH 2.3M was prepared by dissolving 90mg of solid NaOH in 1ml of water while degassing with N_2. A solution of Ketoprofen (10Mm) was prepared in a vial by diluting 25mg of Ketoprofen in 10ml of water and was solubilized by adding 150µl of the previously-prepared NaOH solution together with 30µl Sodium Silicate. The mixture was degassed for 10 minutes. Simultaneously, a 10mM $BaCl_2$ solution was prepared and was also degassed. Following, 0.5ml of the Ketoprofen-Silicate solution was mixed with the same amount of $BaCl_2$ solution (0.5ml) and 2µl of Triton X-100, and the final solution was degassed for 5 minutes.

Then, 20µl of the degassed Ketoprofen-Sodium Silicate-$BaCl_2$ solution were placed in the irradiation cell made by two slides of glass separated by a two layers of adhesive tape as spacers and covered with Baysilone-Paste (GE Bayer Silicones) to have a shielded environment. The current intensity was adjusted at 0.1 and 0.9 A to observe the process induced by two different light intensities. The acquisition was performed for one hour, focusing with the 10X objective and receiving a frame every 10 seconds. After one hour, images with 60X magnification (objective Olympus UPLFLN, 60X) were also collected for the observation of the morphology of the biomorphs.

5 Supporting Videos:

01 Growth of CaCO3 under UV spot irradiation (shown in figure 2) lateral size 1.31x0.88 mm^2 total duration 1200 s

02 Enlargement of 01 size 0.18x0.18 mm^2 total duration 1200 s (shown in figure 2)

03 Growth of biomorph under UV light irradiation at 1.3 mW/mm^2 (shown in figure 4a) 0.39x0.28 total duration mm 7500 s.

04 Growth of biomorph under UV light irradiation at 0.1 mW/mm^2 (shown in figure 4b) 0.20x0.13 total duration 800 s.

References

[1] S. V. Babenko, P. S. Kuznetsova, N. E. Polyakov, A. I. Kruppa, T. V. Leshina, *J. Photochem. Photobiol. A Chem.* **2020**, *392*, 112383.

[2] W. Kashihara, J. Takeyama, T. Suzuki, *J. Photochem. Photobiol. A Chem.* **2020**, *399*, 112623.

[3] K. S. Pérez, A. Moreno, *Crystals* **2019**, *9*, DOI 10.3390/cryst9020067.

[4] Y. Zhao, Y. Xie, S. Yan, Y. Dong, *Cryst. Growth \& Des.* **2009**, *9*, 3072–3078.

[5] T. Nishio, K. Naka, *J. Cryst. Growth* **2015**, *416*, 66–72.

[6] T. Nishio, K. Naka, *J. Cryst. Growth* **2015**, *419*, 79–87.

[7] C. Martínez, S. Vilariño, M. I. Fernández, J. Faria, M. L. Canle, J. A. Santaballa, *Appl. Catal. B Environ.* **2013**, *142–143*, 633–646.

[8] M. Montalti, A. Credi, L. Prodi, T. Candolfi, *Handbook of Photochemistry - 3rd Edition*, **2006**.
